# Physical Culture

**Published:** 1876-07

**Authors:** 


### PHYSICAL CULTURE.

To sedentary persons, violent, sudden, and fitful exercise is always injurious, and such are gymnastic performances.

The exercise of the student should be regular, gentle, deliberate, always stopping short of felt fatigue.

One hour's joyous walk with a cheerful friend, in street, or field, or woodland, will never fail to do a greater and more unmixed good, than double the time in the most scientifically conducted gymnasium in the world.

There are individual cases where the gymnasium is of the most undeniable benefit, but the masses would be the better for having nothing to do with them.

A million times better recipe than the gymnasium for sedentary persons is:

Eat moderately and regularly of plain, nourishing food, well prepared. Spend two or three hours every day in the open air, regardless of all weathers, in moderate untiring activities.

Elsewhere the JOURNAL OF HEALTH teaches that "to derive the highest benefit from exercise as a means of health, it should be in the open air, moderate, continuous, and having an object in view, beside that of the mere exercise itself, which shall be agreeable, interesting, and encouragingly remunerative in a pecuniary point of view.

That, if gymnasiums are founded, they should always be under the immediate direction, control and supervision of those who are thoroughly versed in anatomy and physiology, not merely theoretically but practically.

These positions will scarcely be dissented from by educated physicians, or by any person who has that kind of common-sense which is derived from extended practical observation. Your correspondents, in their strictures on the sentiments of the JOURNAL OF HEALTH, deal somewhat in epithets; that is their taste; it would have answered a better purpose to have employed the same space in explaining how and why muscular exercise is beneficial. The thoughtful reader would then have been able to form a more correct opinion as to the philosophy and the value of physical culture than from any thing the Editor has yet seen in the newspapers on that subject.

Among the anatomical and physiological facts, received the world over, hence requiring no proof here, are the following:

"No two important organs of the body can be called into energetic action at the same time without injury to both, because one organ in high functional action attracts the nervous and sanguineous fluids from the other organs of the system, and any attempt to change the direction of the current suddenly, is always injurious. Hence the ill results to man and beast of active exercise or working, immediately before or after a meal. For the same reason, violent exercise immediately before or after severe study, or after long rest, is always, and under all circumstances, pernicious to the organ of the brain and to the muscular organs.

"Nourishment, repair, growth, strength, all are derived from the blood. If the flow of blood is cut off from any part of the body, that part begins to die on the instant. A steady natural flow of pure blood to a part keeps it in a living, healthful condition. If the flow is increased, but still steady, there is a proportional increase in the vigor of that part. If the supply of blood is very rapid, the ultimate globules or cells are deposited more rapidly than steady nature can receive them, and they are lost or broken, and are passed out of the system as waste, represented in the destruction of glassware in a burning building, when there are more persons to hand it out than there are to receive it.

"Every one knows that exercise of the body increases the circulation of the blood. The violent exercise in gymnasiums, as almost, if not universally conducted hitherto, produces a violent flow of blood, of nutrient particles to the various muscles which are brought into most active exercise, and being carried thither faster than they can be taken up, unmixed harm is the result. Life-long disablements and even deaths have resulted from gymnastic performances and other violent exercises; as of the little girl, not long ago reported, who died in consequence of her ambition to skip a rope a certain number of times without stopping; of race-horses dying on the track; and minor forms of injuries, down to the feeling of soreness of the whole body the day after some unusual exercise; and with which almost every one is familiar.

'Thus it is that the sudden, violent, fitful, exhaustive exercises of ordinary gymnasiums are unwise, hurtful, dangerous. To derive from muscular exertion a high degree of health and manly vigor, it should be moderate, continuous, regular, in the open air, and furthermore, should be pleasantly remunerative beyond the mere benefits of the exercise itself. None of these conditions are fulfilled in gymnasiums as generally conducted hitherto. Physical culture is not objected to, but the manner of it. To exercise wisely, the stüdent and all sedentary persons should begin in moderation, to be gradually increased in its intensity, and as gradually diminished, and in all cases should be left off before any feeling of very great fatigue is experienced, most especial care being taken to cool off very slowly indeed.

